# Supplementary material for: Haplotype and Haplotype-Environment Interaction Analysis Revealed Roles of SPRY2 for NSCL/P among Chinese Populations
Source: Int J Environ Res Public Health. 2019 Feb 15;16(4):557. doi: 10.3390/ijerph16040557 (PMC6406689; doi:10.3390/ijerph16040557)
Supplement: Supplementary file 1 [file ijerph-16-00557-s001.pdf]

International Journal of
Environmental Research and Public Health

MDPI

Supplementary Material

Haplotype and Haplotype-Environment Interaction Analysis Revealed Roles of SPRY2 for NSCL/P among Chinese Populations

Ren Zhou, Mengying Wang, Wenyong Li, Siyue Wang, Hongchen Zheng, Zhibo Zhou, Yonghua Hu, Jing Li, Tao Wu *, Hongping Zhu * and Terri H. Beaty

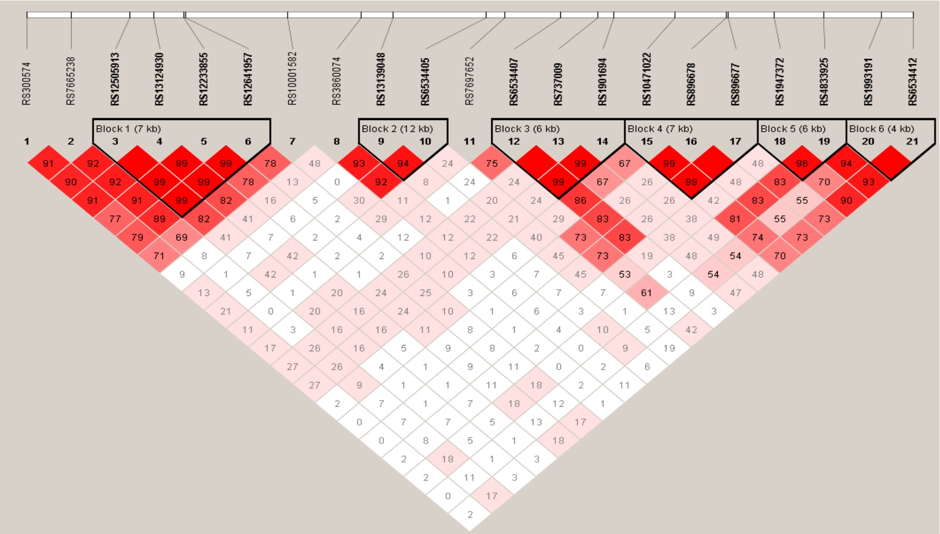

Figure S1. LD plot for single nucleotide polymorphisms (SNPs) in *SPRY1*.

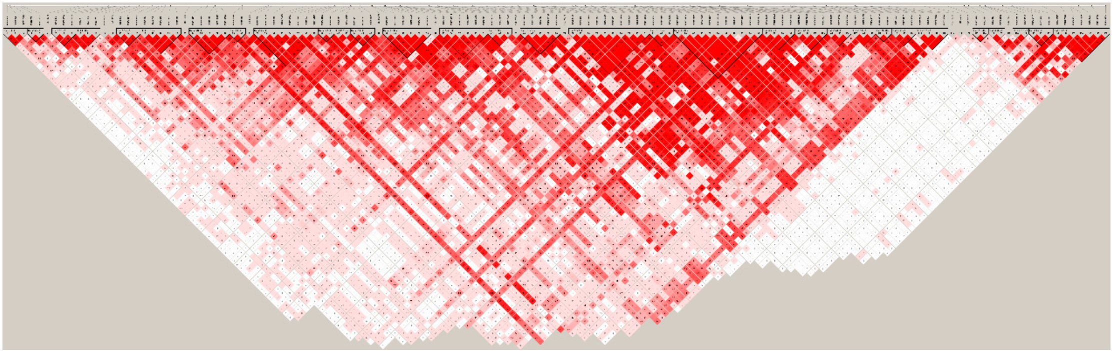

Figure S2. LD plot for single nucleotide polymorphisms (SNPs) in *SPRY2*.

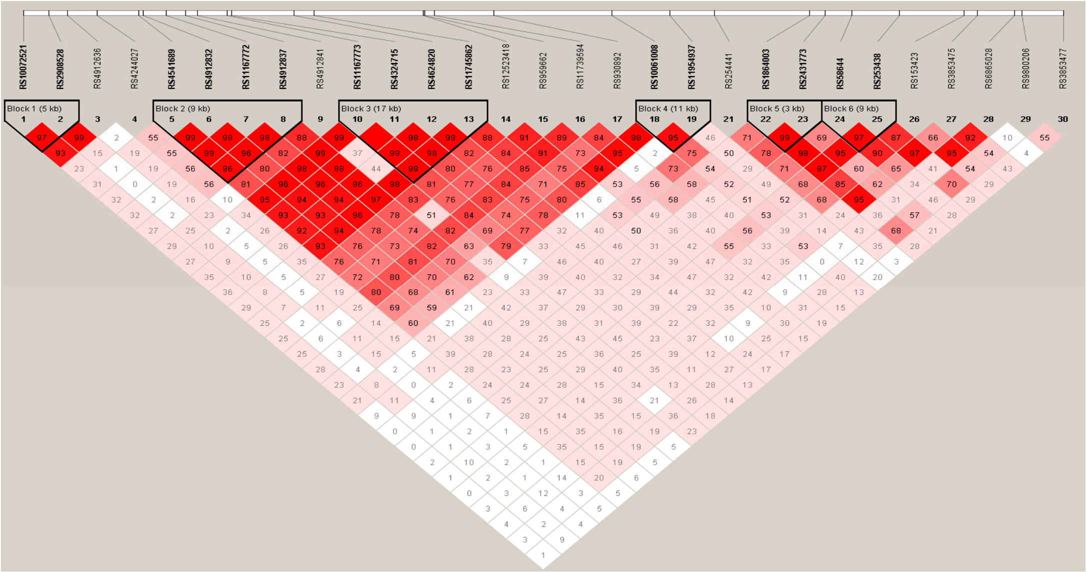

Figure S3. LD plot for single nucleotide polymorphisms (SNPs) in *SPRY4*.

Table S1. 190 single nucleotide polymorphisms (SNPs) included in analysis among 806 Chinese NSCL/P trios.

CHR	SNP	Location [a]	Gene	Minor Allele	MAF [b]
4	RS300574	124543188	SPRY1	C	0.462
4	RS7665238	124549182	SPRY1	G	0.391
4	RS12505913	124556972	SPRY1	G	0.385
4	RS13124930	124560156	SPRY1	G	0.385
4	RS12233855	124564167	SPRY1	T	0.404
4	RS12641957	124564365	SPRY1	T	0.398
4	RS10001582	124578020	SPRY1	C	0.328
4	RS3860074	124587820	SPRY1	G	0.164
4	RS13139048	124592095	SPRY1	A	0.282
4	RS6534405	124604453	SPRY1	A	0.100
4	RS7697652	124607010	SPRY1	A	0.202
4	RS6534407	124614474	SPRY1	G	0.268
4	RS737009	124619446	SPRY1	G	0.208
4	RS1901694	124621473	SPRY1	G	0.209
4	RS10471022	124629718	SPRY1	G	0.436
4	RS896678	124636601	SPRY1	T	0.369
4	RS896677	124636766	SPRY1	C	0.369
4	RS1947372	124642941	SPRY1	A	0.231
4	RS4833925	124649554	SPRY1	T	0.176
4	RS1993191	124657292	SPRY1	A	0.219
4	RS6534412	124661439	SPRY1	A	0.182
5	RS10072521	141608589	SPRY4	A	0.253
5	RS2908528	141613645	SPRY4	T	0.284

5	RS4912636	141617242	SPRY4	G	0.460
5	RS4244027	141623150	SPRY4	T	0.484
5	RS4541689	141631376	SPRY4	G	0.216
5	RS4912832	141632275	SPRY4	A	0.214
5	RS11167772	141636740	SPRY4	C	0.213
5	RS4912837	141640648	SPRY4	T	0.215
5	RS4912841	141642948	SPRY4	T	0.112
5	RS11167773	141648809	SPRY4	C	0.327
5	RS4324715	141649691	SPRY4	T	0.276
5	RS4624820	141661972	SPRY4	A	0.281
5	RS11745862	141665914	SPRY4	T	0.340
5	RS12523418	141687409	SPRY4	A	0.324
5	RS959662	141687790	SPRY4	G	0.293
5	RS11739594	141689747	SPRY4	A	0.318
5	RS930892	141701429	SPRY4	G	0.321
5	RS10061008	141724696	SPRY4	A	0.242
5	RS11954937	141736105	SPRY4	G	0.227
5	RS450988	141738972	SPRY4	A	0.423
5	RS254441	141744840	SPRY4	C	0.461
5	RS1864003	141763720	SPRY4	C	0.246
5	RS2431773	141766790	SPRY4	G	0.338
5	RS58644	141772011	SPRY4	G	0.330
5	RS253438	141781400	SPRY4	A	0.250
5	RS153423	141794129	SPRY4	A	0.314
5	RS3853475	141796799	SPRY4	A	0.404
5	RS6865028	141804032	SPRY4	T	0.141
5	RS9800206	141805508	SPRY4	C	0.412
5	RS3853477	141813688	SPRY4	A	0.236
13	RS348008	79702503	SPRY2	C	0.474
13	RS348005	79705046	SPRY2	T	0.126
13	RS2117906	79709010	SPRY2	G	0.400
13	RS370787	79714566	SPRY2	T	0.202
13	RS9574614	79717326	SPRY2	G	0.153
13	RS1755254	79772282	SPRY2	G	0.220
13	RS722023	79781647	SPRY2	C	0.375
13	RS9574630	79801739	SPRY2	C	0.187
13	RS1928573	79807944	SPRY2	T	0.106
13	RS4728	79808787	SPRY2	C	0.246
13	RS504122	79809526	SPRY2	A	0.460
13	RS541731	79817713	SPRY2	C	0.443
13	RS9531050	79821830	SPRY2	A	0.231
13	RS9545409	79822349	SPRY2	T	0.201
13	RS496932	79831609	SPRY2	A	0.407

13	RS9545412	79836100	SPRY2	G	0.191
13	RS9669948	79844324	SPRY2	G	0.128
13	RS486467	79850613	SPRY2	C	0.315
13	RS502700	79853964	SPRY2	C	0.488
13	RS534870	79857208	SPRY2	G	0.408
13	RS533932	79862299	SPRY2	T	0.486
13	RS556140	79866960	SPRY2	G	0.410
13	RS1215930	79880945	SPRY2	A	0.445
13	RS531545	79893644	SPRY2	T	0.420
13	RS1999494	79898506	SPRY2	A	0.411
13	RS558366	79904872	SPRY2	T	0.420
13	RS2149909	79906110	SPRY2	C	0.420
13	RS9318686	79910614	SPRY2	C	0.410
13	RS9601390	79932505	SPRY2	A	0.127
13	RS9601391	79932825	SPRY2	C	0.451
13	RS9635033	79956028	SPRY2	T	0.437
13	RS7997361	79958104	SPRY2	T	0.373
13	RS1930336	79960942	SPRY2	T	0.156
13	RS2151197	79973128	SPRY2	T	0.452
13	RS13378796	79995156	SPRY2	G	0.240
13	RS1954504	80004574	SPRY2	A	0.238
13	RS9318707	80018185	SPRY2	G	0.357
13	RS1361921	80022675	SPRY2	G	0.249
13	RS9545469	80023085	SPRY2	G	0.376
13	RS9545470	80024512	SPRY2	C	0.205
13	RS1984294	80033985	SPRY2	G	0.421
13	RS2096347	80038567	SPRY2	A	0.243
13	RS2876759	80039485	SPRY2	T	0.420
13	RS7318611	80041591	SPRY2	A	0.177
13	RS7997421	80045918	SPRY2	C	0.159
13	RS2154130	80048171	SPRY2	C	0.419
13	RS17072871	80059099	SPRY2	C	0.362
13	RS9574691	80059655	SPRY2	C	0.432
13	RS7321702	80059964	SPRY2	C	0.409
13	RS9531095	80067462	SPRY2	A	0.216
13	RS1105073	80071409	SPRY2	A	0.269
13	RS954771	80071778	SPRY2	T	0.409
13	RS7989054	80072814	SPRY2	A	0.406
13	RS1772587	80081891	SPRY2	C	0.499
13	RS9574697	80085151	SPRY2	A	0.340
13	RS10507911	80087302	SPRY2	T	0.134
13	RS1772573	80087616	SPRY2	A	0.158
13	RS1772569	80092087	SPRY2	C	0.161

13	RS1176297	80102089	SPRY2	T	0.144
13	RS9565596	80115492	SPRY2	G	0.341
13	RS1582191	80116794	SPRY2	C	0.234
13	RS1176318	80124165	SPRY2	T	0.487
13	RS1176319	80124231	SPRY2	G	0.487
13	RS1417832	80126337	SPRY2	C	0.239
13	RS1176280	80139383	SPRY2	A	0.388
13	RS1176283	80141795	SPRY2	A	0.315
13	RS1417839	80156166	SPRY2	T	0.209
13	RS863712	80158202	SPRY2	G	0.377
13	RS11619199	80170849	SPRY2	G	0.171
13	RS1341476	80182436	SPRY2	A	0.369
13	RS2794235	80187216	SPRY2	C	0.447
13	RS2759243	80195640	SPRY2	A	0.445
13	RS2876761	80202592	SPRY2	A	0.377
13	RS9531108	80218134	SPRY2	C	0.139
13	RS1341473	80218335	SPRY2	A	0.376
13	RS1176270	80218517	SPRY2	A	0.445
13	RS2794251	80229654	SPRY2	T	0.441
13	RS1832799	80235409	SPRY2	A	0.417
13	RS2759236	80237545	SPRY2	T	0.440
13	RS1853300	80255743	SPRY2	A	0.224
13	RS1114275	80259140	SPRY2	T	0.224
13	RS9545554	80268618	SPRY2	G	0.494
13	RS9545555	80274474	SPRY2	C	0.380
13	RS7329886	80284521	SPRY2	G	0.456
13	RS1358977	80286861	SPRY2	A	0.142
13	RS4638453	80305658	SPRY2	C	0.381
13	RS9593490	80307544	SPRY2	A	0.153
13	RS7337052	80315897	SPRY2	G	0.153
13	RS2329172	80317380	SPRY2	A	0.367
13	RS11149181	80321497	SPRY2	C	0.140
13	RS9593494	80328278	SPRY2	A	0.153
13	RS4885741	80331770	SPRY2	A	0.379
13	RS7998402	80353564	SPRY2	G	0.295
13	RS10507913	80357315	SPRY2	T	0.154
13	RS12854744	80363396	SPRY2	G	0.140
13	RS7320320	80371081	SPRY2	T	0.187
13	RS12857748	80372538	SPRY2	G	0.154
13	RS9601485	80373443	SPRY2	T	0.188
13	RS4267202	80377954	SPRY2	G	0.262
13	RS4408423	80378221	SPRY2	A	0.155
13	RS17071498	80388718	SPRY2	C	0.380

13	RS2503378	80397373	SPRY2	C	0.234
13	RS2478223	80400235	SPRY2	A	0.232
13	RS2503380	80402508	SPRY2	C	0.389
13	RS7336590	80427695	SPRY2	G	0.387
13	RS9601506	80428819	SPRY2	G	0.387
13	RS4536348	80460925	SPRY2	C	0.213
13	RS11616409	80469691	SPRY2	A	0.210
13	RS4344612	80472867	SPRY2	G	0.244
13	RS12867395	80491749	SPRY2	G	0.288
13	RS9593517	80492078	SPRY2	G	0.298
13	RS4145077	80504746	SPRY2	T	0.360
13	RS1333417	80508388	SPRY2	T	0.489
13	RS9545588	80513537	SPRY2	G	0.358
13	RS1360632	80532770	SPRY2	G	0.173
13	RS1360631	80532801	SPRY2	A	0.173
13	RS7322700	80547295	SPRY2	G	0.125
13	RS7328506	80547913	SPRY2	C	0.163
13	RS9318729	80564866	SPRY2	C	0.248
13	RS1537442	80571474	SPRY2	C	0.394
13	RS1855259	80600689	SPRY2	A	0.491
13	RS2329177	80623977	SPRY2	G	0.392
13	RS1333404	80639832	SPRY2	G	0.226
13	RS2026514	80644047	SPRY2	A	0.387
13	RS1333421	80655152	SPRY2	A	0.451
13	RS7324138	80660001	SPRY2	C	0.361
13	RS981698	80660337	SPRY2	A	0.359
13	RS12854616	80660902	SPRY2	C	0.297
13	RS9318743	80677414	SPRY2	A	0.206
13	RS9545617	80685676	SPRY2	A	0.499
13	RS7325892	80686898	SPRY2	T	0.262
13	RS1333411	80693350	SPRY2	A	0.375
13	RS9574799	80716279	SPRY2	G	0.112
13	RS1287526	80734028	SPRY2	G	0.330
13	RS9565642	80742866	SPRY2	G	0.108
13	RS1287530	80743554	SPRY2	A	0.175
13	RS1478875	80751833	SPRY2	T	0.495
13	RS1010390	80764540	SPRY2	G	0.388
13	RS1146958	80778298	SPRY2	A	0.321

[a] Based on National Center for Biotechnology Information Human Genome build 36.1.[b] MAF, minor allele frequency.

Table S2. The 5-SNP haplotypes in SPRY genes showing significant association with the risk of NSCL/P or among 806 Chinese trios after Bonferroni correction [1].

SNP Combination	Haplotype	Gene	Frequency (%)	*p*-value
rs4728-rs504122-rs541731-rs9531050-rs9545409	A-A-T-A-G	*SPRY2*	3.0	1.69×10^{-5}
rs504122-rs541731-rs9531050-rs9545409-rs496932	A-T-A-G-A	*SPRY2*	2.5	2.92×10^{-5}
rs541731-rs9531050-rs9545409-rs496932-rs9545412	T-A-G-A-A	*SPRY2*	4.8	3.96×10^{-5}
rs1772587-rs9574697-rs10507911-rs1772573-rs1772569	C-A-T-A-C	*SPRY2*	0.4	4.81×10^{-5}
rs9545412-rs9669948-rs486467-rs502700-rs534870	A-G-A-T-G	*SPRY2*	4.2	1.21×10^{-4}
rs9531050-rs9545409-rs496932-rs9545412-rs9669948	A-G-A-A-G	*SPRY2*	4.4	1.38×10^{-4}
rs954771-rs7989054-rs1772587-rs9574697-rs10507911	C-G-C-A-T:	*SPRY2*	0.2	1.67×10^{-4}
rs7989054-rs1772587-rs9574697-rs10507911-rs1772573	G-C-A-T-A	*SPRY2*	0.2	1.75×10^{-4}

[1] *p*-value threshold= 2.6×10^{-4}.

© 2019 by the authors. Submitted for possible open access publication under the terms and conditions of the Creative Commons Attribution (CC BY) license (http://creativecommons.org/licenses/by/4.0/).
